# How Does a Face Mask Impact Speech Perception?

**DOI:** 10.3390/healthcare10091709

**Published:** 2022-09-07

**Authors:** Il-Joon Moon, Mini Jo, Ga-Young Kim, Nicolas Kim, Young-Sang Cho, Sung-Hwa Hong, Hye-Yoon Seol

**Affiliations:** 1 Department of Otolaryngology-Head & Neck Surgery, Samsung Medical Center, Sungkyunkwan University School of Medicine, Seoul 06351, Korea; 2 Hearing Research Laboratory, Samsung Medical Center, Seoul 06351, Korea; 3 Department of Molecular Biology, Cell Biology, and Biochemistry, Brown University, Providence, RI 02912, USA; 4 Department of Otolaryngology-Head & Neck Surgery, Samsung Changwon Hospital, Sungkyunkwan University School of Medicine, Changwon 51353, Korea; 5 Medical Research Institute, Sungkyunkwan University School of Medicine, Suwon 16419, Korea

**Keywords:** hearing loss, speech perception, mask, COVID-19

## Abstract

Face masks are mandatory during the COVID-19 pandemic, leading to attenuation of sound energy and loss of visual cues which are important for communication. This study explores how a face mask affects speech performance for individuals with and without hearing loss. Four video recordings (a female speaker with and without a face mask and a male speaker with and without a face mask) were used to examine individuals’ speech performance. The participants completed a listen-and-repeat task while watching four types of video recordings. Acoustic characteristics of speech signals based on mask type (no mask, surgical, and N95) were also examined. The availability of visual cues was beneficial for speech understanding—both groups showed significant improvements in speech perception when they were able to see the speaker without the mask. However, when the speakers were wearing the mask, no statistical significance was observed between no visual cues and visual cues conditions. Findings of the study demonstrate that provision of visual cues is beneficial for speech perception for individuals with normal hearing and hearing impairment. This study adds value to the importance of the use of communication strategies during the pandemic where visual information is lost due to the face mask.

## 1. Introduction

Hearing loss, as the name indicates, is the loss of hearing and it can be caused by various factors, such as noise exposure and ototoxic drugs. Depending on one’s hearing characteristics, hearing loss is typically managed by hearing devices, such as hearing aids (HAs) and cochlear implants. A HA is a sound amplifying device that is programmed by a hearing healthcare professional. The HA optimization process involves one’s audiometric data as well as communication needs [1]. For example, while individuals with a quiet lifestyle may not need more than one program for the HAs, those with an active lifestyle may need one for a quiet environment and one for a noisy environment. Aural rehabilitation with cochlear implants is performed in a similar way. In addition to these factors, it has been well documented in literature that both the auditory and visual systems are integral parts of communication. A representative example is the McGurk effect [2]. Here, mismatching auditory ([ba]) and visual ([ga]) signals were perceived as [da] to individuals with NH, suggesting a role of the visual system in speech perception [2]. Many experimental studies have reported the audiovisual integration process for communication [3,4,5,6,7,8,9]. Seol et al. (2021) investigated how immersing patients in the virtual space (VS) of virtual reality technology could influence their speech-in-noise test performance [9]. A total of 30 normal hearing individuals and 25 individuals with hearing loss were asked to listen to and repeat sentences used by the Korean version of the Hearing in Noise Test (K-HINT) speech-in-noise test under three conditions (conventional K-HINT, VS on PC, and VS head-mounted display). The results showed that providing visual cues in the VS led to an improvement in speech performance for all groups, while significantly impacting speech performance between normal and hearing loss groups under all test conditions. In Taitelbaum-Swead and Fostick (2016), the developmental effects of context and modalities—auditory or visual—on audiovisual integration were investigated from the ages 4 to 80 [3]. Participants were instructed to listen to speech stimuli belonging to either of two context conditions (nonsense or meaningful words) for each modality condition (auditory, visual, or audiovisual modalities) then to immediately repeat each word after hearing it. Results suggested that development impacts audiovisual speech perception, with the lowest speech perception accuracy in both audiovisual and auditory modalities occurring at ages 4–5 and 65–80. Children (ages 4–5 and 8–9) also had significantly lower speech perception accuracy than adults (ages 20 and above).

With the recent pandemic, the importance of visual cues has been heightened even more. The use of face masks and social distancing have been mandated, leading to communication challenges. Face masks, as multi-layered fabrics, can reduce sound energy, especially mid-to-high frequency [10,11]. In Bottalico et al. (2020), students with normal hearing (NH) listened to 400 consonant–nucleus–consonant words mixed with a speech-shaped noise. The students completed an online speech recognition test in which they listened to the stimuli and typed what they heard. The results showed that the fabric mask had the greatest sound attenuation (4.2 dB) followed by N95 (2.9 dB) and surgical masks (2.3 dB). On top of the reduced sound energy issue, individuals also lose visual cues due to the face mask covering the mouth. The fabric mask negatively affected speech intelligibility the most; speech intelligibility was decreased by 16%, 13%, and 12% with the fabric, N95, and surgical masks, respectively. Goldin et al. (2020) also investigated how speech signals were influenced by medical masks using a head and torso simulator in four conditions: no mask, simple mask, N95 mask 1, and N95 mask 2 [11]. The results revealed that the N95 masks degraded the acoustic signal by approximately 12 dB which was the most amount of sound attenuation. The simple mask attenuated sounds by about 3 to 4 dB. Mendel et al. (2008) used a surgical mask to examine its impact on speech understanding [12]. In this study, a total of 30 participants with NH and hearing impairment (HI) listened to sentences from the Connected Speech Test and repeated them back to the tester in four conditions (without a mask in quiet, without a mask in noise, with a mask in quiet, and with a mask in noise). The results showed that those with HI experienced significant difficulty understanding speech in noise. Spectral analyses of the speech stimuli also showed a significant difference between the with and without mask conditions. Although several studies have examined the influence of face masks on speech intelligibility and conducted spectral analyses, there is a lack of sufficient data to determine the frequency where the maximum amount of sound attenuation occurs. This study explores how a face mask affects speech understanding in quiet for individuals with and without hearing loss as well as frequencies that are affected the most by a face mask.

## 2. Materials and Methods

### 2.1. Participants

Participants who met the following criteria were enrolled in the study: (a) native Korean speakers and (b) adults (19 years and above). With the inclusion criteria, a total of 25 adult participants, who were native Korean speakers, were enrolled in the study. The age range of the participants was from 19 to 69 years old. The mean age of the NH group was 24.6 years (SD = 3.3) while the mean age of the hearing impaired (HI) group was 58.0 years (SD = 11.8). Among the 25 participants, 14 of them had NH and 11 had bilateral moderate sensorineural hearing loss with an asymmetry in hearing thresholds below 10 dB across testing frequencies (250, 500, 1000, 2000, 4000, and 8000 Hz). The NH group’s puretone averages were 3.2 dB in the right ear and 2.4 in the left ear. The HI group had puretone averages of 42.5 dB in the right ear and 43.6 dB in the left ear. Individuals who were unable to watch TV at a distance of 1 m and those with middle ear pathology and neurological and mental disorders were excluded from the study. All experimental procedures were approved by Samsung Medical Center’s Institutional Review Board. An informed consent document was obtained from the participants. Informed consent was also obtained from speakers to publish the images in an online publication.

### 2.2. Conventional Puretone Audiometry

Conventional pure-tone audiometry was performed in a sound booth using an AudioStar Pro (Grason-Stadler, Eden Prairie, MN, USA) audiometer and insert earphones.

### 2.3. Video Recordings

**Figure 1:**
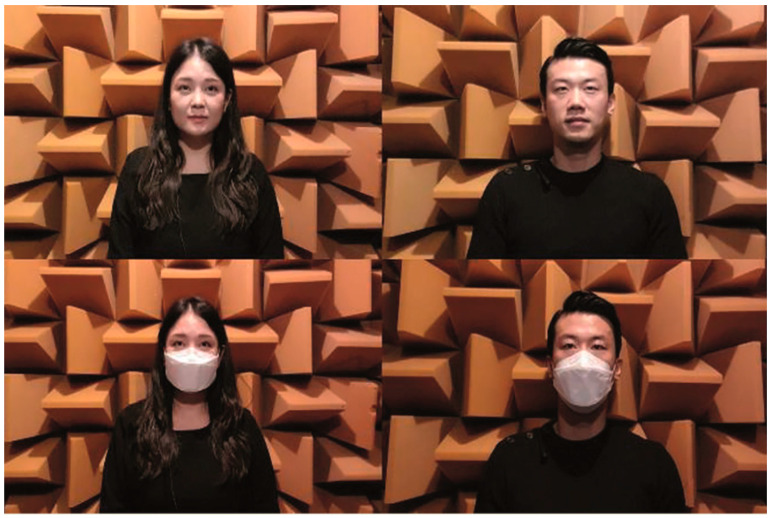
Screenshot of the video recordings used in the study.

A total of four video recordings were created: a female native Korean speaker with and without a face mask and a male native Korean speaker with and without a face mask (Figure 1). The mask used in the study was KF94. The ‘KF’ stands for ‘Korea Filter’ and ‘94′ refers to the 94% filtration efficiency. The KF 94 masks are reported to be equivalent to N95 and FFP2 masks [13,14]. The speakers recorded the Korean Standard Sentences Lists for Adults (KSSL-A) [15]. The KSSL-A consists of 80 sentences and 320 target words; there are 8 lists and each list has 10 sentences and 40 target words. The KSSL-A sentences contained everyday sentences, such as ‘I went to a department store to buy a necklace and a ring’ and ‘Please wait here’. The recordings were then edited using commercial editing tools from Adobe Systems, San Jose, CA, USA: Adobe Premiere Pro and Adobe Audition. In order to reflect the real-world conversations, the recordings were edited in a way that the video recordings of the female and male speakers were alternating. 

### 2.4. Acoustic Measurement of Speech Signals Based on Mask Type

Using the Knowles Electronics Manikin for Acoustic Research (KEMAR), which is a manikin used for audiological research, the sound pressure level of speech signals was measured in three conditions: no mask, surgical mask, and N95. Audio recordings of the videos (List 1 only) were presented through a loudspeaker that was placed 1 m away. The surgical and N95 masks were placed around the loudspeaker for the surgical and N95 conditions, respectively.

### 2.5. Speech Testing

The speech testing was conducted using the video recordings in a semi-anechoic chamber. The participants were asked to repeat target sentences that were presented through a loudspeaker at 50 dBA. The loudspeaker was placed 1 m away from the participant. The number of accurately repeated sentences was calculated to determine percent-correct scores. Using the four video recordings, the participants completed the following test conditions: no mask and no visual cues, no mask and visual cues, mask and no visual cues, and mask and visual cues. The video recordings were displayed on a monitor and the monitor was turned off for the no visual cues condition. The test conditions were randomized for each participant. The testing took an hour and breaks were given as needed.

### 2.6. Statistical Analysis

Using the Statistical Package for the Social Sciences (SPSS) version 26 (IBM Corporation, Armonk, NY, USA), the Wilcoxon-signed rank test was performed to analyze the differences between the mask and unmask conditions as well as no visual cues and visual cues conditions for NH and HI groups. Non-parametric tests were used as our results did not pass the normality test.

## 3. Results

### 3.1. Sound Attenuation Based on Mask Type

**Figure 2:**
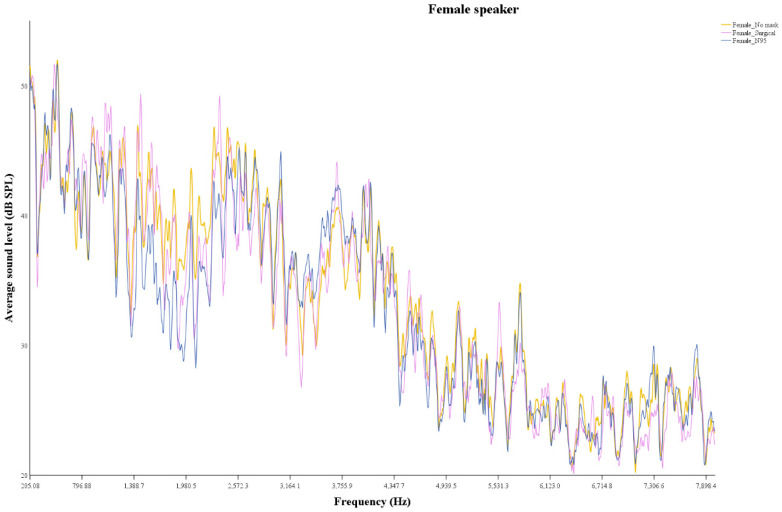
Average sound pressure level for female speaker.

**Figure 3:**
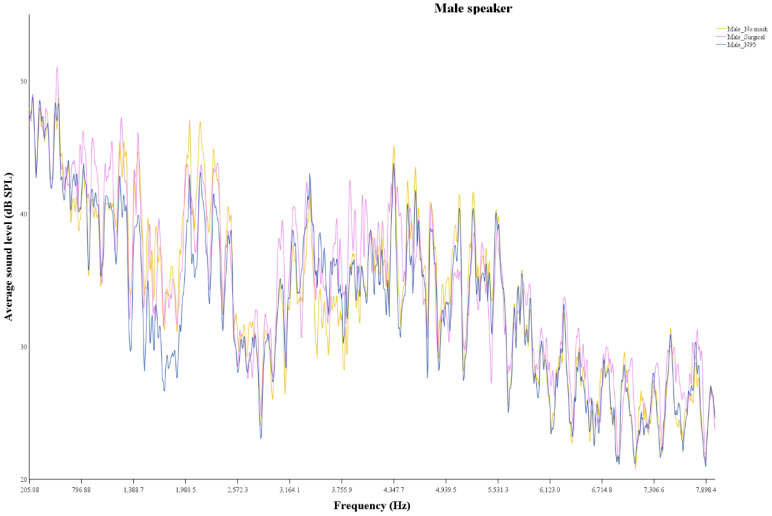
Average sound pressure level for male speaker.

The amount of sound attenuation based on mask type was examined for both speakers (Figure 2 and Figure 3). For the female speaker, the mean sound pressure levels were 33.6, 32.9, and 32.8 dB SPL for the no mask, surgical, and N95 conditions, respectively. Differences of sound pressure levels between the conditions were 0.7 dB SPL (no mask − surgical) and 0.8 dB SPL (no mask − N95). For the male speaker, the mean sound pressure levels were 33.9, 34.5, and 33.1 dB SPL for the no mask, surgical, and N95 conditions, respectively. Differences of sound pressure level between the three masks were −0.6 dB SPL (no mask − surgical) and 0.8 dB SPL (no mask − N95). For both speakers, statistical significance was observed between no mask and surgical (*p* < 0.001) and between no mask and N95 conditions (*p* < 0.001)—when the speakers were wearing the surgical and the N95 mask, the sound pressure levels were significantly reduced. The maximum sound attenuation occurred at 2560 Hz (8.2 dB) between the no mask and the surgical conditions and at 1798 Hz (7.5 dB) between the no mask and the N95 conditions for the female speaker. For the male speaker, the amount of sound attenuation was the greatest at 4593 Hz (7.2 dB) between the no mask and the surgical conditions and at 1816 Hz (6.8 dB) between the no mask and the N95 conditions.

### 3.2. The Impact of Visual Cues on the NH and HI Groups

Differences in the speech performance between the NH and HI groups are illustrated in Table 1. In the no mask condition, significant differences were observed between the no visual and visual cues conditions for both groups (*p* = 0.007 for the NH group and *p* = 0.005 for the HI group). However, no significant differences were observed between the two conditions for both groups in the mask condition (*p* = 0.120 for the NH group and *p* = 0.391 for the HI group).

### 3.3. The Impact of Face Mask on the NH and HI Groups

The impact of face mask on speech understanding was also investigated (Table 2). In the visual cues condition, significant differences were observed between the unmask and mask conditions (*p* = 0.006 for the NH group and *p* = 0.004 for the HI group) for both groups. Face masks, however, did not have any impact on speech understanding when visual cues were unavailable (*p* = 0.812 for the NH group and *p* = 0.632 for the HI group) for both groups.

## 4. Discussion

In our study, when the speakers were not wearing a face mask, speech recognition improved with the provision of visual information regardless of the presence of hearing loss. However, when the speakers were wearing the face mask, provision of visual cues did not have any significant impact on speech performance for both groups. In a case where visual cues were not available, wearing a face mask had a significant impact on speech understanding, but no statistical significance was observed between the mask and no mask conditions when visual cues were available to the participants. The findings are in line with previous studies to some extent that the availability of visual information is beneficial for communication—the participants showed better speech performance even if the speakers were wearing a face mask [3,5,6,9,16,17]. As to sound attenuation, significant differences were observed. The sound pressure level differences ranged from −0.6 to 0.8 dB SPL between the conditions for male and female speakers. The maximum amount of sound attenuation occurred at 2560 (surgical mask) and 1798 Hz (N95 mask) for the female speaker and at 4593 (surgical mask) and 1816 Hz (N95 mask) for the male speaker. All of these frequencies fall under the frequency range (500–4000 Hz) that is essential for speech intelligibility [18]. The findings are in line with pre-existing studies to a large extent that masks attenuate high frequency sounds [11,19].

Findings of this study are meaningful as they add value to the use of communication strategies. As mentioned earlier, the loss of visual cues with the face mask could lead to communication difficulty and in medical settings, in particular, communication challenges could interfere with the delivery of patient-centered care [20,21,22,23,24,25,26]. For example, Pamungkasih et al. (2019) conducted interviews with patients to explore their acceptance of surgical masks worn by doctors. The study revealed that the clarity of conversations decreases when doctors were wearing a mask and some patients do not always ask their doctors to clarify. This could create a barrier between the patients and the doctors [20,21,24], as well as between healthcare providers [20]. Hence, we, along with other researchers, encourage healthcare professionals to actively use communication strategies, especially when interacting with individuals with hearing loss. Examples of communication strategies include use of clear speech and assistive listening devices, facing the speaker, and gaining attention before starting a conversation [20]. Eby et al. (2020) recommended healthcare professionals to use assistive listening devices, such as a personal sound amplification product to effectively communicate with their patients. With the importance of visual information in mind, it is critical for both the speaker and the listener to face each other and speak slowly and clearly to communicate with a better signal-to-noise ratio [20]. Marler and Ditton (2020) highlighted that providing introductions and explanations is essential. Additionally, the authors recommended putting emphasis on body language to express empathy and understanding [24]. As for limitations, for the current study, only a single type of mask as well as a listening condition (quiet) were utilized. Including a noise condition, different types of masks and various topics of videos (not just speech testing sentences) could reflect the real-world setting better and make visual cues even more essential for accurate speech recognition. Subsequent studies with larger sample size and more variety in participant characteristics and masks are currently underway. Examining the primary mode of communication (speaking and sign language) would allow us to compare the impact of the primary mode of communication on speech perception during the pandemic. In addition, since there are features that can enhance speech levels on mobile phones, it would be worth investigating the feature’s impact on speech perception when the visual cues are lost due to face masks. Performing real-ear measurements would aid us in exploring how much sound energy is decreased by different types of masks and how this reduction in sound energy is related to speech performance in various listening conditions.

## Figures and Tables

**Table 1:** Statistical analysis of the groups’ speech performance in the no visual cues and visual cues conditions (* *p* < 0.05).

Group	Conditions	Provision of Visual Cues Median (Inter Quartile Range)		*p*
		No	Yes	
NH	No mask	80 (70–90)	100 (90–100)	0.007 *
	Mask	80 (67.5–90)	90 (77.5–100)	0.120
HI	No mask	20 (20–30)	80 (40–90)	0.005 *
	Mask	10 (10–60)	30 (10–60)	0.391

**Table 2:** Statistical analysis of the groups’ speech performance in the mask and no mask conditions (* *p* < 0.05).

Group	Conditions	Use of Face Mask Median (Inter Quartile Range)		*p*
		No	Yes	
NH	No visual cues	100 (90–100)	90 (77.5–100)	0.006 *
	Visual cues	80 (70–100)	80 (67.5–90)	0.812
HI	No visual cues	80 (40–90)	30 (10–60)	0.004 *
	Visual cues	20 (20–30)	10 (10–60)	0.632

## Data Availability

The data supporting the findings of this study are available from the corresponding author, H.Y.S., upon reasonable request. The data were not publicly available because of ethical considerations.
